# Role of Mitochondrial Genome Mutations in Pathogenesis of Carotid Atherosclerosis

**DOI:** 10.1155/2017/6934394

**Published:** 2017-07-25

**Authors:** Margarita A. Sazonova, Vasily V. Sinyov, Anastasia I. Ryzhkova, Elena V. Galitsyna, Zukhra B. Khasanova, Anton Yu Postnov, Elena I. Yarygina, Alexander N. Orekhov, Igor A. Sobenin

**Affiliations:** ^1^Russian Cardiology Research and Production Complex, Moscow 121552, 15a, 3rd Cherepkovskaya street, Moscow 121552, Russia; ^2^Institute of General Pathology and Pathophysiology, Russian Academy of Medical Sciences, Moscow 125315, 8, Baltiyskaya st., Moscow 125315, Russia; ^3^K.I. Skryabin Moscow State Academy of Veterinary Medicine and Biotechnology-MVA, 23, Skryabina st., Moscow 109472, Russia; ^4^Department of Genetics, Southern Federal University, 105/42, B. Sadovaya st., Rostov-on-Don, 344006, Russia; ^5^Institute for Atherosclerosis Research, 121609, Skolkovo Innovative Centre, Moscow Region, Skolkovo, Novaya st., Moscow, Russia

## Abstract

Mutations of mtDNA, due to their higher frequency of occurrence compared to nuclear DNA mutations, are the most promising biomarkers for assessing predisposition of the occurrence and development of atherogenesis. The aim of the present article was an analysis of correlation of several mitochondrial genome mutations with carotid atherosclerosis. Leukocytes from blood of study participants from Moscow polyclinics were used as research material. The sample size was 700 people. The sample members were diagnosed with “atherosclerosis” on the basis of ultrasonographic examination and biochemical and molecular cell tests. DNA was isolated from blood leukocyte samples of the study participants. PCR fragments of DNA, containing the region of 11 investigated mutations, were pyrosequenced. The heteroplasmy level of these mutations was detected. Statistical analysis of the obtained results was performed using the software package SPSS 22.0. According to the obtained results, an association of mutations m.652delG, m.3336C>T, m.12315G>A, m.14459G>A m.15059G>A with carotid atherosclerosis was found. These mutations can be biomarkers for assessing predisposition to this disease. Additionally, two single nucleotide substitutions (m.13513G>A and m.14846G>A), negatively correlating with atherosclerotic lesions, were detected. These mutations may be potential candidates for gene therapy of atherosclerosis and its risk factors.

## 1. Introduction

In recent years, more and more attention is paid to molecular genetic diagnostics of polygenic multifactorial diseases, including cardiovascular pathologies and atherosclerosis. Atherosclerosis occurs in many men and women of middle age [1, 2]. Meanwhile, atherosclerotic lesions are occurring nowadays in a far higher percentage of young people than before. A search of molecular genetic markers which can be appreciated as early predictors of atherosclerosis is an important task, because it is very hard to recognize atherosclerosis by the existing “classical” clinical methods in the early stages of the disease.

MtDNA mutations, due to their higher frequency of occurrence compared to nuclear DNA mutations, are the most promising biomarkers for assessing predisposition of the occurrence and development of atherogenesis. Each cell of a human organism, depending on the tissue, which it belongs to, has form one to several hundred mitochondria. In each of mitochondria, there are some copies of mitochondrial genome. Mitochondrial genome is characterized by the maternal type of inheritance. Somatic mutations often occur in mtDNA, the expressivity of which notably depends on the heteroplasmy level.

Even though the linkage of autosome mutations with atherosclerosis has been reported [3–6], not much research was devoted so far to analysis of mitochondrial genome defects [7–11]. The majority of such works was devoted to large-scale deletions [8, 12, 13]. In our preliminary study, which focused on an analysis of 42 mitochondrial genome mutations associated with different pathologies, 11 mutations (m.652delG, m.1555G>A, m.3256C>T, m.3336C>T, m.5178C>A, m.652insG, m.15059G>A, m.13513G>A, m.14459G>A, m.14846G>A, and m.12315G>A) linked with atherosclerosis of aorta were detected [14–18]. In the present article, the heteroplasmy level of the detected mtDNA mutations in carotid atherosclerosis was analyzed.

## 2. Materials and Methods

Leukocytes from blood of patients from Moscow polyclinics were used as research material. The sample size was 700 people. Men were older than 40 years, and women were older than 50. The sample members were diagnosed with “atherosclerosis” on the basis of ultrasonographic examination and biochemical and molecular cell tests [19, 20].

There were 700 participants selected for the study, which made up two approximately equal groups:

1. Conventionally healthy patients;
2. Study participants which were at high risk of occurrence and development of atherosclerotic lesions.

The following methods of investigation were used as the following:

1. Isolation of DNA using the method of phenol-chloroform extraction [21–23], developed in our laboratories on the basis of a technology, suggested by Maniatis et al. [24];
2. Polymerase chain reaction (PCR) in order to obtain DNA fragments containing the region of the investigated mutations [17, 18];
3. Pyrosequencing of PCR fragments [25];
4. Analysis of the heteroplasmy level in the investigated mutations using the original quantitative method previously developed by the authors of this article on the basis of pyrosequencing technology [17, 18, 26];
5. Statistical analysis of the obtained results by using the software package SPSS 22.0 [27].

During the statistical analysis of results, Mann–Whitney *U*-test for independent samples and Wilcoxon test for dependent samples were used. The correlation coefficient was determined on the basis of Spearman contingency table analysis and bootstrap analysis. The linear regression method was used to interpret the direction of link between the stage of atherosclerotic lesion and the percentage of heteroplasmy. Factorial regression was used to assess the degree of association of atherosclerotic lesions with the heteroplasmic percentage value of the investigated mutations.

## 3. Results and Discussion

Eleven mitochondrial genome mutations, detected during the analysis of affected by atherosclerosis segments of aortas, were decided to be analyzed in blood cells of 700 study participants from the Moscow region, in which during clinical and ultrasonographic examinations atherosclerotic lesions of carotid arteries were found. The average age of the participants was 64.7 years.

### 3.1. Linkage of mtDNA Mutations with Atherosclerotic Plaques in Carotid Arteries

#### 3.1.1. Detection of Eleven Mitochondrial Mutations

A statistically significant positive correlation of atherosclerotic plaques with the heteroplasmy level mutations m.652delG, m.3336C>T, m.12315G>A, and m.14459G>A and a negative correlation with mutation m.14846G>A (*p* ≤ 0.001) were detected (Table 1).

#### 3.1.2. Heteroplasmy Level of Mutation m.13513G>A

Antiatherogenic effect of allele A at position 13,513 was shown at the heteroplasmy level above 65% in atherosclerotic plaques (*p* ≤ 0.05) (Table 2).

#### 3.1.3. Total Mutational Burden in Atherosclerotic Plaques in Carotid Arteries

As the effect of different mutations is multidirectional, it is necessary to consider the cumulative impact of 11 studied mutations or total mutational burden. This characteristic was assessed in two stages:

1. the construction of logistic regression model (Tables 3–5);
2. the construction of ROC curves (Table 6).

A predictor in the analysis of ROC curves was a probability belonging to one category or another (0 (no atherosclerotic plaques) or 1 (the presence of atherosclerotic plaques of any size)).

**Figure 1:**
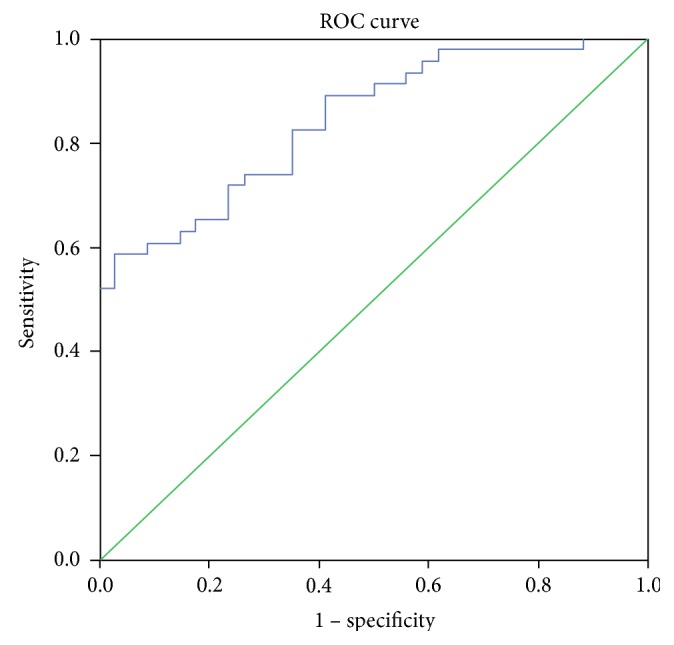
ROC curve for the analysis of total mutation burden of 11 mitochondrial genome mutations as genetic markers of the presence of atherosclerotic plaques in carotid arteries.

On the basis of values of the included variables, with the use of the model, the estimation of probability belonging to a category “0” or “1” for each study participant was performed. The data obtained on the probability, which can be considered as a measure of the relative risk, were used for ROC analysis (Figure 1, Table 6).

According to the data of ROC analysis, the model turned out to be significant. The threshold value of 0.54 was chosen; it corresponded to the sensitivity value of 0.739 and specificity value of 0.735 (*p* ≤ 0.05).

Therefore, the predictive and explanatory ability of the model for total mutation burden in atherosclerotic plaques of any size was significantly higher than that of the models made separately for each mutation. The total mutational burden of 11 investigated mitochondrial genome mutations was associated with 84.2% of atherosclerotic plaques in the carotid arteries in human.

### 3.2. Association of Mutations with the Intima-Media Thickness of Carotid Arteries (IMT CA)

#### 3.2.1. Analysis of 11 Mitochondrial Genome Mutations

According to the statistical data (Table 7), the level of heteroplasmy in blood cells significantly positively correlated with the thickening of the intima-medial layer of carotid arteries for mutations m.12315G>A and m.15059G>A (*p* ≤ 0,05).

A significant negative correlation with the present parameter was found for mutations m.13513G>A and m.14846G>A (*p* ≤ 0.05).

#### 3.2.2. Total Mutational Burden in Thickening of Intima-Medial Layer of Carotid Arteries

Due to the fact that the effect of different mutations on a change of the IMT CA was multidirectional, it was necessary to consider the cumulative impact of 11 studied mutations or total mutational burden. This characteristic was assessed in two stages, as it was in section 3.1.3. At the preliminary stage, a binary logistic regression model was built (Tables 8–10).

**Figure 2:**
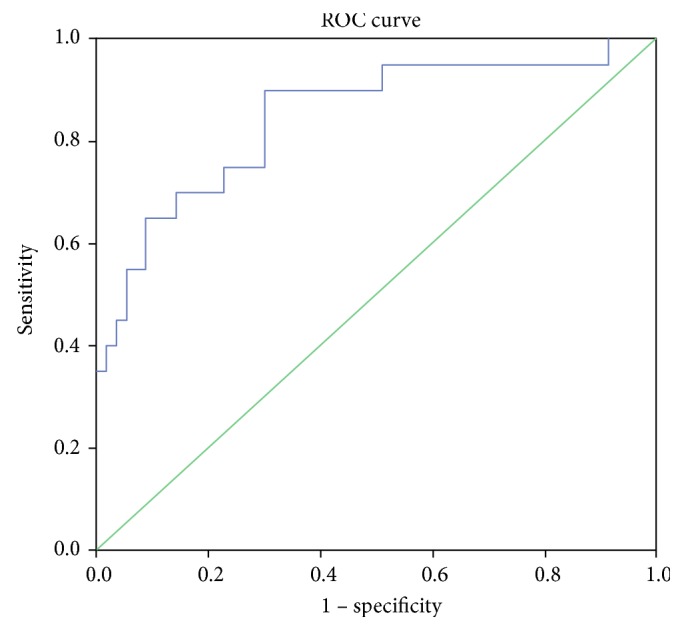
The ROC curve for the analysis of total mutation burden of 11 mitochondrial genome mutations as genetic markers of the presence of thickening of the intima-medial layer of carotid arteries.

Analysis of ROC curves is presented in Figure 2 and Table 11.

On the basis of values of the included variables, the model estimated the probability belonging to a category 0 or 1 for each study participant. The data on the probability, which can be considered as a measure of relative task, were used for the ROC analysis (Figure 2, Table 11).

The area under the curve was 0.849, consequently the model turned out to be significant. Sensitivity was 0.700; specificity was 0.900 (*p* ≤ 0.05).

The evaluation of the predictive and explanatory power of the model for total mutational burden in IMT CA allowed us to consider that the predictive and explanatory power in the used model was significantly higher than that in the models constructed individually for each mutation. Total mutational burden of 11 studied mitochondrial genome mutations was associated with 84.9% of thickening of the intima-medial layer of carotid arteries in humans.

Therefore, an analysis of mtDNA mutations in a large representative sample of 700 study participants, which included patients with atherosclerosis and conventionally healthy individuals, was carried out. The sample members were diagnosed with “atherosclerosis” on the basis of ultrasonographic examination and biochemical and molecular cell tests. Four mutations of the mitochondrial genome, highly significantly linked with the presence of atherosclerotic plaques in patients (m.652delG, m.3336C>T, m.12315G>A, and m.14459G>A) were identified. Two single nucleotide substitutions of mtDNA were significantly linked with the absence of atherosclerotic plaques in individuals (m.13513G>A and m.14846G>A). Additionally, two mitochondrial mutations were detected (m.12315G>A and m.15059G>A), which significantly positively correlated with the thickening of the intima-medial layer of carotid arteries. Two other mutations significantly negatively correlated with the thickening of the intima-medial layer of carotid arteries (m.13513G>A and m.14846G>A). These mutations are localized in MT-RNR1, MT-TL2, MT-ND1, MT-ND6, and MT-CYTB. It suggests an idea of an important role of genes of subunits of respiratory chain enzymes of mitochondria and ribosomal RNA and also an important role of transfer RNA-leucine in atherogenesis processes. A negative correlation of mutations m.13513G>A (MT-ND6) and m.14846G>A (MT-CYTB) with atherosclerosis may indicate that these mutations can lead to enzyme stabilization and can make their work more efficient. The total mutational burden of eleven studied mitochondrial mutations was associated with more than 84% cases of occurrence of atherosclerotic plaques and pathological thickening of the intima-medial layer of carotid arteries, which indicates the high diagnostic value of complex analysis of these mutations in atherosclerosis. Therefore, the most optimal for gene diagnostics of atherosclerosis would be the use of all these eleven investigated mtDNA mutations, as it enables the evaluation of predisposition to atherosclerosis and its early diagnosis in the maximum number of patients.

However, we should acknowledge certain limitations of this study. Although the study was performed in rather large sample of atherosclerotic patients and apparently healthy nonatherosclerotic individuals, it should be noted that using the same samples in whom the described association was originally identified may artificially augment the strength of prediction. With such level of prediction, it may be suggested that mitochondrial mutations are extremely powerful risk factors, but the effect size of these mutations may be probably a lot lower and artificially inflated using the same data. Therefore, replication studies in independent naïve cohorts are necessary to confirm the findings.

It is necessary to mention that although the investigated sample consisted of 700 study participants, the statistical processing of the results included a bootstrap analysis during which the manyfold-increased sample of patients with atherosclerosis was compared with manyfold-increased sample of conditionally healthy donors.

We suppose that in our further studies, in which a much larger sample of study participants will be analyzed, it will not be necessary to resort to bootstrap analysis.

## 4. Conclusion

The importance of detecting mtDNA mutations linked with occurrence and development of atherosclerotic lesions in humans does not admit of doubt due to the fact that the mortality from atherosclerosis is at a very high level.

In the present study, an association of five mutations with carotid atherosclerosis was found (m.652delG, m.3336C>T, m.12315G>A, m.14459G>A m.15059G>A). These mutations can be biomarkers for assessing predisposition to this disease. Additionally, two single nucleotide substitutions (m.13513G>A and m.14846G>A), negatively correlating with atherosclerotic lesions, were detected. These mutations may be potential candidates for gene therapy of atherosclerosis and its risk factors.

The results obtained in this study may be useful to medical geneticists, specializing in the evaluation of the predisposition to atherosclerosis and other vascular diseases and also in the early diagnosis of these pathologies.

## Figures and Tables

**Table 1:** Bootstrap analysis of correlation of individuals' atherosclerotic plaques with the heteroplasmy level of mitochondrial mutations.

Mutation	Correlation coefficient value	Asymptotical significance (2-tailed)
m.652delG	0,464	0,010^∗∗^
m.652insG	−0,319	0,060^∗^
m.1555A>G	−0,163	0,247
m.3256C>T	0,274	0,101^∗^
m.3336T>C	0,593	0,002^∗∗^
m.5178C>A	0,305	0,064^∗^
m.12315G>A	0,612	0,001^∗∗^
m.13513G>A	−0,201	0,187
m.14459G>A	0,605	0,001^∗∗^
m.14846G>A	−0,452	0,010^∗∗^
m.15059G>A	0,116	0,212

^∗∗^Significant correlation of mutations with atherosclerotic plaques (*p* ≤ 0,05); ^∗^Correlation of mutations with atherosclerotic plaques at *p* ≤ 0,1 level of significance.

**Table 2:** Association of 65% heteroplasmy level of allele 13513A with the absence of atherosclerotic plaques in carotid arteries (APCA).

Spearman correlation		65% heteroplasmy level of m.13513G>A	APCA
65% heteroplasmy level of m.13513G>A	Correlation coefficient	1000	−0,204
	Significance (2-tailed)	—	0,054
	Number of valid cases	90	90

APCA	Correlation coefficient	−0,204	1000
	Significance (2-tailed)	0,054	—
	Number of valid cases	90	502

**Table 3:** Summary of a linear regression model of interconnection of mutational burden with atherosclerotic plaque in carotid arteries.

Model	Minus twice the log likelihood	Cox and Snell *R*^2^	Nagelkerke *R*^2^
1	147,273	0,358	0,481^∗^

^∗^The complex of features explains the dispersion of the dependent variable at 48,1%.

**Table 4:** Classification of cases of atherosclerotic plaque association with a total burden of 11 mutations.

Model	Detected cases		Predicted cases		
			Association of atherosclerotic plaques with a total burden of 11 mutations		Percentage of correct predictions
			0,00	1,00	
1	Association of atherosclerotic plaques with a total burden of 11 mutations	0,00	44	24	64,7
		1,00	24	68	73,9
	Total percentage value				70,0^∗^

^∗^The percentage of correctly classified cases was 70%.

**Table 5:** The analysis of included variables and the coefficient of link force and direction.

Analyzed variables							
	Mutations	B	S.E.	Wald	df	Sig.	Exp (B)
Model 1	m.1555A>G	−0,163	0,042	14,952	1	0,000^∗∗^	0,850
	m.3256C>T	0,033	0,051	0,417	1	0,519	1033
	m.14846G>A	−0,026	0,029	0,845	1	0,358	0,974
	m.5178C>A	0,034	0,045	0,560	1	0,454	1034
	m.652delG	0,052	0,022	5761	1	0,016^∗∗^	1054
	m.12315G>A	0,122	0,027	20,958	1	0,000^∗∗^	1130
	m.13513G>A	−0,046	0,017	6951	1	0,008^∗∗^	1047
	m.14459G>A	0,030	0,015	3971	1	0,046^∗∗^	0,970
	m.15059G>A	0,052	0,020	6836	1	0,009^∗∗^	1054
	m.652insG	0,077	0,081	0,901	1	0,343	1080
	m.3336T>C	0,052	0,028	3420	1	0,064^∗^	1054
	Constant	−2384	1308	3321	1	0,068^∗^	0,092

Coefficient B indicates the link direction; ^∗∗^Significant correlation of mutations with atherosclerotic plaques in carotid arteries (*p* ≤ 0,05); ^∗^Correlation of mutations with atherosclerotic plaques at *p* ≤ 0,1 level of significance.

**Table 6:** ROC analysis of interconnection of mutational burden with atherosclerotic plaques in carotid arteries.

Probability of faultless prognosis				
Area under the curve	Standard error	Asymptomatic significance	Asymptomatic confidence interval 95%	
			Lower than 95%	Higher than 95%
0,842	0,030	0,001	0,784	0,900

**Table 7:** Bootstrap analysis of сorrelation of mitochondrial genome mutations with the IMT CA.

Mutation	Correlation coefficient	Asymptotical significance (2-tailed)
m.652delG	0,161	0,206
m.652insG	0,017	0,722
m.1555A>G	0,009	0,865
m.3256C>T	0,329	0,091^∗^
m.3336T>C	0,081	0,383
m.5178C>A	0,318	0,103^∗^
m.12315G>A	0,619	0,001^∗∗^
m.13513G>A	−0,478	0,050^∗∗^
m.14459G>A	0,157	0,236
m.14846G>A	−0,493	0,045^∗∗^
m.15059G>A	0,529	0,028^∗∗^

^∗∗^Significant correlation of mutations with the IMT CA (*p* ≤ 0,05); ^∗^Correlation of mutations with the IMT CA at *p* ≤ 0,1 level of significance.

**Table 8:** Summary of linear regression model of interconnection of mutational burden with IMT CA.

Model	Minus twice the log likelihood	Cox and Snell *R*^2^	Nagelkerke *R*^2^
1	65,002	0,260	0,382^∗^

^∗^The complex of features explains the dispersion of the dependent variable at 38,2%.

**Table 9:** Classification of cases of IMT CA association with a total burden of 11 mtDNA mutations.

Model	Detected cases		Predicted cases		
			Association of IMT CA with a total burden of 11 mutations		Percentage of correct predictions
			0,00	1,00	
1	Association of IMT CA with a total burden of 11 mutations	0,00	55	2	96,5
		1,00	11	9	45,0
	Total percentage value				83,1^∗^

^∗^The percentage of correctly classified cases was 83,1%.

**Table 10:** The analysis of included variables and the coefficient of link force and direction.

Analyzed variables							
	Mutations	B	S.E.	Wald	df	Sig.	Exp(B)
Model 1	m.1555A>G	−0,177	0,079	5017	1	0,025^∗^	0,838
	m.3256C>T	0,098	0,071	1916	1	0,166	1103
	m.14846G>A	−0,108	0,051	4483	1	0,034^∗^	0,898
	m.5178C>A	0,079	0,078	1024	1	0,312	0,924
	m.652delG	0,006	0,036	0,032	1	0,857	0,994
	m.12315G>A	0,027	0,030	0,806	1	0,369	1028
	m.13513G>A	−0,023	0,023	1001	1	0,317	1023
	m.14459G>A	0,018	0,026	0,463	1	0,496	0,982
	m.15059G>A	0,026	0,023	1273	1	0,259	1027
	m.652insG	−0,187	0,156	1439	1	0,230	1205
	m.3336T>C	0,028	0,028	1036	1	0,309	1028
	Constant	1350	1913	0,498	1	0,480	3858

Coefficient B indicated the link direction; ^∗^Significant correlation of mutations with atherosclerotic plaques in carotid arteries (*p* ≤ 0,05).

**Table 11:** ROC analysis of interconnection of mutational burden with IMT CA.

Probability of faultless prognosis				
Area under the curve	Standard error	Asymptomatic significance	Asymptomatic confidence interval 95%	
			Lower than 95%	Higher than 95%
0,849	0,055	0,001	0,742	0,956
